# Falls in Parkinson's disease: the impact of disease progression, treatment, and motor complications

**DOI:** 10.1590/1980-5764-DN-2021-0019

**Published:** 2022-04-29

**Authors:** Danielle Pessoa Lima, Samuel Brito de-Almeida, Janine de Carvalho Bonfadini, Alexandre Henrique Silva Carneiro, João Rafael Gomes de Luna, Madeleine Sales de Alencar, Antonio Brazil Viana-Júnior, Pedro Gustavo Barros Rodrigues, Isabelle de Sousa Pereira, Jarbas de Sá Roriz-Filho, Manoel Alves Sobreira-Neto, Pedro Braga-Neto

**Affiliations:** 1 Universidade Federal do Ceará, Departamento de Clínica Médica, Divisão de Neurologia, Fortaleza CE, Brazil.; 2 Universidade Federal do Ceará, Departamento de Clínica Médica, Divisão de Geriatria, Fortaleza CE, Brazil.; 3 Universidade de Fortaleza, Faculdade de Medicina, Fortaleza CE, Brazil.; 4 Universidade Federal do Ceará, Hospital Universitário Walter Cantídio, Unidade de Pesquisa Clínica, Fortaleza CE, Brazil.; 5 Universidade Unichristus, Faculdade de Medicina, Fortaleza CE, Brazil.; 6 Universidade Estadual do Ceará, Centro de Ciência da Saúde, Fortaleza CE, Brazil.

**Keywords:** Accidental Falls, Parkinson Disease, Gait, Acidentes por Quedas, Doença de Parkinson, Marcha

## Abstract

The prevalence of Parkinson's disease (PD) tends to increase worldwide in the coming decades. Thus, the incidence of falls is likely to increase, with a relevant burden on the health care system.

**Objective:**

The objective of this study was to evaluate clinical factors and drug use associated with falls in PD patients.

**Methods:**

We conducted a cross-sectional study at the Movement Disorders outpatient clinic of a tertiary hospital in Northeast Brazil. We performed structured interviews to collect sociodemographic and clinical data. Functional capacity was assessed using the Schwab and England Activities of Daily Living Scale and the modified Hoehn and Yahr Staging Scale. We divided the study sample into non-fallers (no falls) and fallers (≥1 fall), and non-recurrent (≤1 fall) and recurrent fallers (>1 fall).

**Results:**

The study population comprised 327 PD patients (48% women), with a mean age of 70 years. The mean disease duration was 9.9±6.9 years. The most prevalent comorbidities were depression (47.2%), hypertension (44.0%), and type 2 *diabetes mellitus* (21.5%). The logistic regression analysis revealed that hallucinations, amantadine, and catechol-*O*-methyltransferase inhibitors (entacapone) were independently associated with falls in PD patients. Also, hallucinations, dyskinesia, and the use of amantadine were independently associated with recurrent falls.

**Conclusions:**

Health care providers play an essential role in fall prevention in PD patients, particularly by identifying older adults experiencing dyskinesia and visual hallucinations. Prospective studies should investigate the use of amantadine as a risk factor for falls in PD patients.

## RESUMO.

Estima-se aumento na prevalência da doença de Parkinson (DP) em todo o mundo nas próximas décadas. Dessa forma, espera-se também aumento na incidência de quedas e seu impacto no sistema de saúde.

**Objetivo:**

O objetivo deste estudo foi avaliar fatores clínicos e medicamentos associados a quedas em pacientes com DP.

**Métodos:**

Trata-se de um estudo observacional transversal, realizado no ambulatório de Distúrbios do Movimento de hospital terciário no Brasil. Os dados sociodemográficos e clínicos foram coletados por meio de entrevista estruturada. A capacidade funcional foi avaliada pela Escala de Atividades de Vida Diária de Schwab e England e o estadiamento por Hoehn e Yahr modificado. A amostra foi dividida em não caidores (0 quedas) e caidores (≥1 queda) e não caidores recorrentes (≤1 queda) e caidores recorrentes (>1 queda). A informação sobre o número de quedas nos últimos seis meses foi confirmada com familiares e cuidadores.

**Resultados:**

A população do estudo foi de 327 pacientes (48% mulheres), com idade média de 70 anos e duração média da doença de 9,9±6,9 anos. As comorbidades mais prevalentes foram depressão (47,2%), hipertensão (44%) e *diabetes mellitus* tipo 2 (21,5%). A análise de regressão logística revelou que alucinações visuais, uso de amantadina e uso de entacapona foram independentemente associadas a quedas. Alucinações visuais, discinesia e uso de amantadina foram independentemente associados a quedas recorrentes neste estudo.

**Conclusões:**

Os profissionais de saúde desempenham um papel importante na prevenção de quedas em pacientes com DP, principalmente idosos que apresentam discinesia e alucinações visuais. Estudos prospectivos da amantadina devem ser realizados para investigar sua associação com quedas em pacientes com DP.

## INTRODUCTION

Parkinson's disease (PD) is the second most common neurodegenerative disorder^1^. It is a complex disease that causes motor and non-motor symptoms and impairs functionality^2^. People with PD require regular medical and multiprofessional evaluations for function adjustments, rehabilitation, and management of complications. The annual risk of falls in PD ranges from 45 to 68%^3^. Notably, some parkinsonian factors could predict fall risk, including orthostatic hypotension, freezing of gait, disease severity, and postural instability^4^. Recurrent falls are an issue in the life of PD patients and suggest disease progression^3^. The definition of recurrent falls comprises more than one fall in a certain period^5^ and allows discriminating people who happened to fall from those with an increased intrinsic risk for falls^3^.

Underlying factors associated with recurrent falls in PD patients are different from those of the general population^5^. Risk factors for falls may be intrinsic or extrinsic. Intrinsic factors include physiological changes related to age, balance and gait alterations, visual and hearing impairment, and the presence of comorbidities. Extrinsic factors include environmental risks, such as insufficient lighting and slippery floor, risk behaviors, and behaviors related to activities of daily living^6^.

The prevalence of PD tends to increase worldwide in the coming decades^1^. Thus, the incidence of falls is likely to increase with a relevant burden on the health care system, considering their consequences. Falls may result in deaths, decreased mobility, and lower quality of life^7^. It is a complex issue, with a long way for effective prevention and treatment^5^. Previous studies have found an association between the use of certain drugs and increased risk of falls in older people^8–10^. The most effective prevention programs have multidimensional strategies to reduce falls, including careful regular review of medication use^11^. Neurological consultations of PD patients have often focused on prescribing antiparkinsonian drugs and addressing motor symptoms^12^. However, it is necessary to actively address factors associated with fall risk during a routine neurological consultation to prevent future falls. In this sense, neurologists should be aware of fall risk and review the medications prescribed to their patients. Many studies focus on falls in older people. However, few studies have addressed falls in PD, focusing on medications associated with increased risk of falls. Thus, the purpose of this study was to characterize the clinical features of PD patients and assess variables related to the occurrence of falls and recurrent falls in the previous 6 months.

## METHODS

### Study design and participants

We conducted a cross-sectional study to evaluate consecutive PD patients who attended the Movement Disorders outpatient clinic of Hospital Universitário Walter Cantidio (HUWC), Fortaleza (Ceará), Northeast Brazil. We followed the Strengthening the Reporting of Observational Studies in Epidemiology (STROBE) reporting guidelines for cross-sectional studies. Participants were recruited from January 2018 to August 2019. Diagnosis of PD was confirmed when they fulfilled the clinical diagnostic criteria by the Movement Disorders Society and the U.K. Parkinson's Disease Society Brain Bank. We excluded the patients with severe PD, according to the modified Hoehn and Yahr (HY) Scale for PD Staging (HY-5).

### Clinical evaluation

We evaluated the patients during regular face-to-face consultations. Our team comprised two neurology residents, one internal medicine resident, one geriatrics resident, two neurologists, and one geriatrician previously trained to evaluate PD patients. Potential confounders were minimized by the previous training to address these patients. We used a structured questionnaire to collect sociodemographic and clinical information, including age, sex, disease duration, family history of PD, marital status, history of hypertension, diabetes, cardiac insufficiency, peripheral arterial disease, cancer, chronic obstructive pulmonary disease^13^, chronic kidney disease, orthostatic hypotension, stroke^14^, dementia, mild neurocognitive disorder (15), epilepsy, hip fracture, depression, bipolar affective disorder^15^, osteoporosis, osteoarthritis, and urgent urinary incontinence.

We also collected information on antiparkinsonian drug treatments, including l-DOPA (l-DOPA/carbidopa, l-DOPA/benserazide, and sustained-release formulation of l-DOPA), catechol-*O*-methyltransferase (COMT) inhibitors (entacapone), monoamine oxidase B (MAO-B) inhibitors (rasagiline), amantadine, and dopamine agonists (pramipexole). To compare different doses of antiparkinsonian medications, we adopted the levodopa equivalent dose (LED) according to a systematic review by Tomlinson et al.^16^.

Moreover, we collected information on the number of drugs used by each participant. We used the Schwab and England Activities of Daily Living (SE ADL) Scale to assess the ability to perform ADL and the modified HY scale for PD staging^17^. We evaluated all patients during the “on” phase. We collected any information on complications of antiparkinsonian treatments, including visual hallucinations, dyskinesias, and motor fluctuations, by consulting patients, family, caregivers, and clinical records. In case of difficulties obtaining information with the patients (e.g., cognitive impairment), we collected the data by interviewing their family or caregivers. The interview was followed by a physical examination, in which clinical changes not perceived by the patients or their family could be recognized.

### Assessment of falls

A fall was defined as an event that results in a person coming to rest unintentionally on the floor or another lower level without any cause (e.g., violent behavior, assaults, or car or bike accidents) or precipitant (syncope or epilepsy)^18^. A faller was defined as the patient who had at least one fall and a non-faller who did not have any fall in the past 6 months. A non-recurrent faller was defined as the individual who had none or one fall. A recurrent faller was defined as an individual who had two or more falls^3^. We evaluated the number of falls in the past 6 months before the medical evaluation.

### Statistical analysis

We calculated the mean, standard deviation, and median for each continuous variable. We compared non-fallers (0 falls) and fallers (≥1 fall), as well as non-recurrent (≤1 fall) and recurrent fallers (>1 fall). We used the Shapiro-Wilk test to assess the normality distribution of data. We compared the demographic and clinical characteristics between the groups using the Mann-Whitney U test for continuous variables and the X^2^ test or Fisher's exact test for categorical variables. We constructed two separate multivariate logistic regression models using a forward stepwise procedure to assess the relationship between the study variables (fallers vs. non-fallers and non-recurrent fallers vs. recurrent fallers). Fallers and recurrent fallers were considered as dichotomous-dependent variables (yes or no) in the regression models.

**Figure 1:**
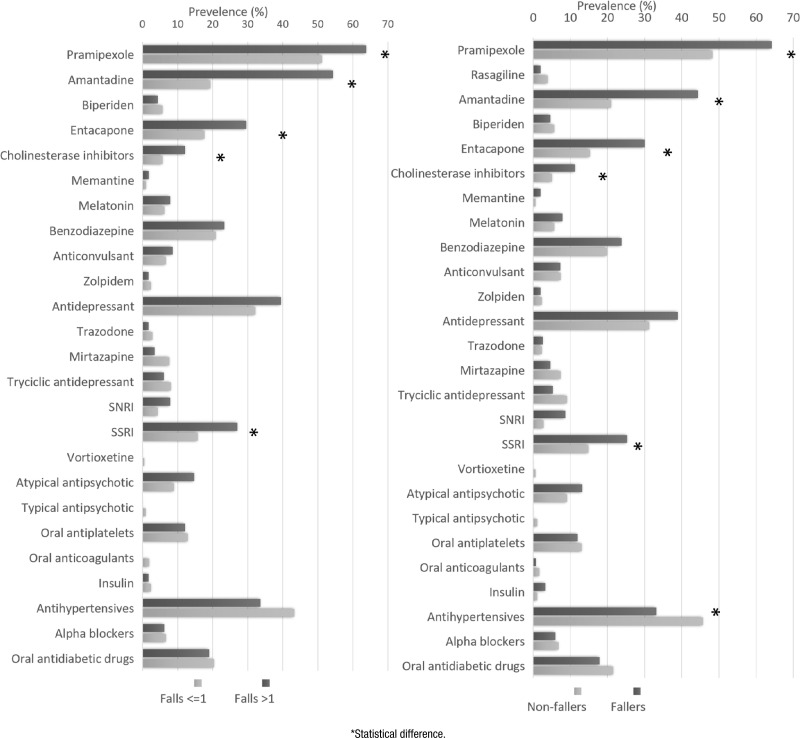
Frequency between fallers and medication use.

After conducting a univariate analysis including the variables of interest (Tables 1 and 2, and Figure 1), we included those with p<0.05 in the multivariate regression analysis (Tables 3 and 4). For the multivariate regression analyses, we used binary logistic regression analysis with a forward stepwise method. A detailed description of these analyses with the interactions of the models and the inputs and outputs of variables are available in Supplementary Materials 1 and 2. We used the variance inflation factor to verify multicollinearity in the independent variables. Statistical analyses were performed using the JAMOVI statistics package (version 0.9).

**Table 1:** Sociodemographic and clinical characteristics of fallers and non-fallers among patients with Parkinson's disease.

	Fallers	Non-fallers	p-value	OR (95%CI)
Age (years)	70 (60–78)	71 (60–78)	0.761^a^	–
Male sex, n (%)	86 (57.0)	84 (47.7)	0.096^b^	1.45 (0.94–2.24)
Family history of PD, n (%)	32 (22.1)	29 (16.7)	0.222^b^	1.42 (0.81–2.48)
Sleep complaints, n (%)	104 (69.3)	114 (65.1)	0.423^b^	1.21 (0.76–1.93)
Motor fluctuations, n (%)	95 (65.1)	92 (52.9)	0.027^b^	1.66 (1.06–2.61)
Dyskinesia, n (%)	75 (51.0)	45 (25.9)	<0.001^b^	2.99 (1.87–4.77)
Hallucinations, n (%)	49 (34.3)	26 (15.8)	<0.001^b^	2.79 (1.62–4.80)
Hypertension, n (%)	57 (37.7)	87 (49.4)	0.034^b^	0.62 (0.40–0.97)
Type 2 DM, n (%)	30 (19.9)	40 (23.0)	0.495^b^	0.83 (0.49–1.42)
Congestive heart failure, n (%)	2 (1.3)	6 (3.4)	0.294^b^	0.38 (0.08–1.90)
Coronary artery disease, n (%)	7 (4.6)	11 (6.3)	0.515^b^	0.72 (0.27–1.92)
Peripheral artery disease, n (%)	0 (0.0)	2 (1.1)	0.501^c^	0.23 (0.01–4.81)
Chronic venous insufficiency, n (%)	3 (2.0)	4 (2.3)	>0.999^c^	0.87 (0.19–3.96)
Active cancer, n (%)	3 (2.0)	3 (1.8)	>0.999^c^	1.14 (0.23–5.71)
Previous cancer, n (%)	3.5 (5)	3.4 (6)	>0.999^c^	0.94 (0.28–3.14)
COPD, n (%)	2 (1.3)	3 (1.7)	>0.999^c^	0.77 (0.13–4.67)
Chronic kidney disease, n (%)	2 (1.3)	3 (1.7)	>0.999^c^	0.77 (0.13–4.68)
Orthostatic hypotension, n (%)	33 (28.9)	33 (28.0)	0.868^b^	1.05 (0.59–1.86)
Previous stroke, n (%)	11 (7.3)	7 (4.1)	0.204^b^	1.87 (0.70–4.94)
Dementia, n (%)	32 (21.3)	27 (15.4)	0.169^b^	1.49 (0.84–2.62)
Mild cognitive impairment, n (%)	15 (10.3)	9 (5.4)	0.109^b^	2.00 (0.85–4.71)
Epilepsy, n (%)	3 (2)	5 (2.8)	0.731^c^	0.71 (0.17–3.01)
Hip fracture, n (%)	3 (2)	4 (2.3)	>0.999^c^	0.88 (0.19–3.99)
Depression, n (%)	79 (52.7)	75 (42.6)	0.070^d^	1.50 (0.97–2.32)
Bipolar disorder, n (%)	1 (0.7)	2 (1.1)	>0.999^b^	0.58 (0.05–6.46)
Osteoporosis, n (%)	15 (10.3)	21 (13.1)	0.452^b^	0.76 (0.38–1.54)
Osteoarthritis, n (%)	28 (19.9)	27 (17.4)	0.590^b^	1.17 (0.65–2.11)
Urinary incontinence, n (%)	65 (43.6)	54 (30.9)	0.018^b^	1.73 (1.10–2.74)
Walking aids, n (%)	35 (25)	26 (15.8)	0.044^b^	1.78 (1.01–3.14)
Motor physical therapy, n (%)	22 (16.3)	20 (12.3)	0.331^b^	1.38 (0.72–2.66)
Hoehn and Yahr stage	3 (2–4)	2.64±1.15 (2.25)	0.002^a^	–
SE ADL score	80 (50–90)	80 (50–90)	0.007^a^	–
Disease duration (years)	10 (6–17)	2.25 (2–3)	<0.001^a^	–

Data expressed as percentages and medians (25th–75th).

a Mann-Whitney U test;

b Pearson's X^2^ test;

c Fisher's exact test.

PD: Parkinson's disease; SE ADL: Schwab and England Activities of Daily Living scale; DM: *diabetes mellitus*; COPD: chronic obstructive pulmonary disease; SNRIs: serotonin–norepinephrine reuptake inhibitors; SSRIs: selective serotonin reuptake inhibitors; OR: *Odds Ratio*; 95%CI: 95% confidence interval. Bold values denote a statistically significant difference.

**Table 2:** Sociodemographic and clinical characteristics of occasional and recurrent fallers among patients with Parkinson's disease.

	Falls			
	>1	≤1	p-value	OR (95%CI)
Age (years)	70 (63–76)	70 (60–78)	0.728^a^	–
Male gender, n (%)	64 (55.2)	106 (50.2)	0.393^b^	1.22 (0.77–1.92)
Family history of PD, n (%)	22 (19.8)	39 (18.8)	0.817^b^	1.07 (0.60–1.92)
Sleep complaints, n (%)	81 (70.4)	137 (65.2)	0.340^b^	1.27 (0.78–2.07)
Motor fluctuations, n (%)	78 (69.6)	109 (52.4)	0.003^b^	2.08 (1.28–3.39)
Dyskinesia, n (%)	68 (60.2)	52 (25.0)	<0.001^b^	4.53 (2.78–7.40)
Hallucinations, n (%)	41 (37.3)	34 (17.2)	<0.001^b^	2.87 (1.68–4.89)
Hypertension, n (%)	46 (39.7)	98 (46.4)	0.237^b^	0.76 (0.48–1.20)
Type 2 DM, n (%)	22 (19.0)	48 (23.0)	0.401^b^	0.79 (0.45–1.38)
Congestive heart failure, n (%)	2 (1.7)	6 (2.9)	0.717^c^	0.60 (0.12–3.00)
Coronary artery disease, n (%)	4 (3.4)	14 (6.7)	0.223^b^	0.50 (0.16–1.56)
Peripheral artery disease, n (%)	0 (0.0)	2 (1.0)	0.539^c^	0.35 (0.02–.745)
Chronic venous insufficiency, n (%)	3 (2.6)	4 (1.9)	0.703^c^	1.36 (0.30–6.19)
Active cancer, n (%)	2 (1.7)	4 (1.9)	>0.999^c^	0.89 (0.16–4.91)
Previous cancer, n (%)	4 (3.5)	7 (3.5)	>0.999^c^	1.00 (0.29–3.51)
COPD, n (%)	3 (2.6)	2 (1.0)	0.352^c^	2.76 (0.45–16.7)
Chronic kidney disease, n (%)	2 (1.7)	3 (1.4)	>0.999^c^	1.20 (0.20–7.31)
Orthostatic hypotension, n (%)	26 (29.5)	40 (27.8)	0.772^b^	1.09 (0.61–1.96)
Previous stroke, n (%)	9 (7.8)	9 (4.4)	0.204^b^	1.84 (0.71–4.78)
Dementia, n (%)	28 (24.1)	31 (14.8)	0.037^b^	1.83 (1.03–3.23)
Mild cognitive impairment, n (%)	14 (12.5)	10 (5.0)	0.017^b^	2.71 (1.16–6.33)
Epilepsy, n (%)	2 (1.8)	6 (2.9)	0.717^c^	0.61 (0.12–3.06)
Hip fracture, n (%)	3 (2.6)	4 (1.9)	0.700^c^	1.39 (0.31–6.33)
Hearing impairment, n (%)	12 (10.5)	14 (6.8)	0.247^b^	1.61 (0.72–3.60)
Visual impairment, n (%)	10 (8.8)	9 (4.4)	0.108^b^	2.11 (0.83–5.37)
Depression, n (%)	62 (53.4)	92 (43.8)	0.095^b^	1.47 (0.93–2.32)
Bipolar disorder, n (%)	1 (0.9)	2 (0.9)	>0.999^c^	0.91 (0.08–10.13)
Osteoporosis, n (%)	11 (9.8)	25 (13.0)	0.414^b^	0.73 (0.35–1.55)
Osteoarthritis, n (%)	21 (19.3)	34 (18.2)	0.817^b^	1.07 (0.59–1.96)
Urinary incontinence, n (%)	51 (44.7)	68 (32.4)	0.028^b^	1.69 (1.06–2.70)
Walking aids, n (%)	29 (27.4)	32 (16.1)	0.019^b^	1.97 (1.11–3.48)
Motor physical therapy, n (%)	14 (13.3)	28 (14.6)	0.768^b^	0.90 (0.45–1.80)
Hoehn and Yahr stage	3.0 (2.5–4.0)	2.3 (2.0–3.0)	<0.001^a^	–
SE ADL score	75 (50–90)	80 (60–90)	0.007^a^	–
Disease duration (years)	10.00 (6.00–17.00)	7.00 (4.00–11.00)	<0.001^a^	–

Data expressed as percentages and medians (25th–75th).

a Mann-Whitney U test;

b Pearson's X^2^ test;

c Fisher's exact test.

PD: Parkinson's disease; SE ADL: Schwab and England Activities of Daily Living scale; DM: *diabetes mellitus*; COPD: chronic obstructive pulmonary disease; SNRIs: serotonin–norepinephrine reuptake inhibitors; SSRIs: selective serotonin reuptake inhibitors; OR: *Odds Ratio*; 95%CI: confidence interval. Bold values denote a statistically significant difference.

**Table 3:** Multivariate logistic regression model.

	OR (95%CI)	p-value
Amantadine use	2.81 (1.63–4.85)	<0.001
Hallucinations	2.49 (1.35–4.61)	0.004
COMT inhibitor/entacapone use	2.03 (1.10–3.72)	0.023

Dependent variable: fall in the past 6 months (yes or no). OR: *Odds Ratio*; 95%CI: confidence interval; COMT: catechol-*O*-methyltransferase.

**Table 4:** Multivariate logistic regression model.

	OR (95%CI)	p-value
Hallucinations	3.19 (1.71–5.94)	<0.001
Amantadine use	3.13 (1.60–6.12)	0.001
Dyskinesia	2.20 (1.14–4.23)	0.019

Dependent variable: fallers (yes or no). OR: *Odds Ratio*; 95%CI: 95% confidence interval.

### Ethics

The local ethics committee approved the study (registration number: 91075318.1.0000.5045). All participants gave their written informed consent before the collection of data. We conducted all procedures by following the ethical standards of the human experimentation committee and the principles of the Declaration of Helsinki.

## RESULTS

We included 327 patients in the analysis (48% women). The mean age was 70 years old, and the mean disease duration was 9.9±6.9 years. Over half of them (201; 62.2%) were married. Notably, 61 (19.1%) participants had a family history of PD. The most common clinical manifestations associated with PD were sleep disorders (67.1%), motor fluctuations (58.4%), urinary incontinence (36.7%), and orthostatic hypotension (28.4%). The most prevalent comorbidities were depression (47.2%), hypertension (44%), and type 2 *diabetes mellitus* (21.5%). Sociodemographic variables were not significantly associated with fallers (Table 1). Figure 1 illustrates the frequency of medication use among fallers and recurrent fallers.

The participants were sorted into two groups: fallers and non-fallers in the first analysis and recurrent fallers and non-fallers in the second analysis. In the logistic regression models, we included the clinical symptoms that were substantially related to the occurrence of falls in the previous 6 months: motor fluctuations (odds ratio [OR]=1.66, p=0.027), dyskinesia (OR=2.99, p<0.001), hallucinations (OR=2.79, p<0.001), hypertension (OR=0.62, p=0.034), urinary incontinence (OR=1.73, p=0.018), and walking aids (OR=1.78, p=0.044). Regarding the scale variables, the significant ones were HY stage (p=0.002), SE ADL score (p=0.007), and disease duration (p<0.001).

Table 1 and Figure 1 show the variables significantly associated with falls in the univariate analysis. The following clinical symptoms were shown to be categorical variables substantially related to recurrent falls: dyskinesia (OR=4.53, p<0.001), hallucinations (OR=2.87, p<0.001), mild cognitive impairment (OR=2.71, p=0.017), motor fluctuations (OR=2.08, p=0.003), walking aids (OR=1.97, p=0.019), dementia (OR=1.83, p=0.037), and urinary incontinence (OR=1.69, p=0.028). Regarding the scale variables, the significant ones were HY stage (p<0.001), SE ADL score (p=0.007), and disease duration (p<0.001). Table 3 and Figure 1 show the variables significantly associated with recurrent fallers in the univariate analysis. These variables were included in the logistic regression models (Table 4).

## DISCUSSION

We showed that amantadine use, visual hallucinations, and entacapone use were significantly associated with falls. In the multivariate analysis, we found an association of recurrent falls with hallucinations, amantadine use, and dyskinesias. Amantadine use was the independent variable and most strongly associated with falls, and hallucinations were most strongly associated with recurrent falls. Fallers and recurrent fallers are likely to have more severe symptoms and present dyskinesias, requiring more antiparkinsonian drugs^9,19^. Moreover, they were likely to have more motor fluctuations and receive entacapone to improve these symptoms^20^. Also, patients with advanced PD have more hallucinations that are associated with cognitive decline^21^.

Dyskinesia encompasses abnormal and involuntary movements, and its treatment is often challenging^16^. In a systematic review, Manson et al.^22^. concluded that dyskinesia in PD ranges from 40% to 50% within 5 years of treatment, and this rate may increase to 50–75% within 10 years of treatment.^22^ Most patients have mild dyskinesia without functional impairment. This symptom may be improved with adjustments in dopaminergic drug treatment, reflecting a more cautious use of levodopa^23^, once amantadine's side effects include blurred vision, dizziness, hallucinations, confusion, urinary disturbances, constipation, orthostatic hypotension, peripheral edema, and dry mouth^23^, which could partially account for the increased fall risk. Also, amantadine is a drug frequently used for the treatment of dyskinesia. In this study, we found an association of amantadine use with falls and recurrent falls. Amantadine potentially causes anticholinergic effects^24^, and its anti-dyskinetic effects are transient. Thomas et al.^25^ reported that amantadine reduced dyskinesia, but from 3 to 8 months of treatment. Amantadine elimination is through renal clearance, and toxicity is more common in elderly patients with renal dysfunction. Thus, it is necessary to measure nitrogenous bases before prescribing this medication^24^. Individualized risk-to-benefit assessment should form a part of the decision on maintaining this drug treatment for dyskinesia in PD patients.

Dyskinesia was associated with recurrent falls in our study. In a 1-year prospective study, Rudziñska et al.^26^ compared 106 PD patients against 55 age-matched controls. They reported a rate of fall of 54% in PD patients compared with 18% in controls. In another study, 3.6% of falls in PD patients were due to severe dyskinesia^26^. In a 6-month prospective study conducted with 64 PD patients without dementia or severe comorbidities, Lamont et al.^27^ indicated that a prevalence of 54% experienced near falls or falls. The authors found that dyskinesia, defined as the occurrence of scores ≥1 in the Unified Parkinson's Disease Rating Scale item-32, was the strongest predictor of future or near falls^27^. Involuntary movements that interfere with gait can explain the link between dyskinesia and repeated falls. Indeed, gait is impaired in patients with severe dyskinesia and advanced PD^23^.

Visual hallucinations are frequent in PD, and their prevalence ranges from 30 to 50% in cross-sectional studies^28^. A hypocholinergic status may be involved in the pathophysiology of visual hallucinations^29^ once cholinergic deficit causes impaired attention and visual hallucinations^30^. Hallucinations were significantly associated with falls and recurrent falls in our study, supporting the hypothesis that acetylcholine reduction is related to hallucinations, which in turn are associated with impaired attention and increased risk of fall^29^.

The cholinergic system of subcortical regions in the striatum, thalamus, and cerebellum plays a substantial role in mobility. Executive function is essential for cognition and the cognitive control of gait and balance^31^. Cholinergic activity in the thalamus, originating from the pedunculopontine nucleus (PPN), is essential for gait regulation and is implicated in gait impairment in PD. Some studies have evidenced cholinergic dysfunction by using positron emission tomography in PD patients, experiencing falls compared to non-fallers^32^.

Hallucinations in PD probably involve a cholinergic neuronal loss and a loss of dopaminergic neurons. Cholinergic dysfunction causes attention deficits and impairment in executive functions. Hence, gait and balance may no longer be compensated by attentional control, thus increasing the risk of falls^33^.

In this study, we also found an association between COMT inhibitors and falls. In a case–control study conducted in U.S. veterans with hip fractures, French et al.^34^ showed that the association between antiparkinsonian drug use and falls was four times higher in cases compared with controls. However, it is unlikely to determine whether this association is due to the disease or the medicines used to treat it.

Considering that the main side effects of COMT inhibitors are nausea, diarrhea, vomiting, and postural hypotension^35^, the increased risk of falls due to drug use may be due to these distress symptoms. Patients expressing discomfort due to these symptoms were 2–5 times more likely to fall. However, long-term studies are needed to confirm the use of COMT inhibitors as a predictor of falls^36^.

In a case-control study that included patients with parkinsonism and PD, Vestegaard et al.^37^ evaluated the association between the incidence of fracture and antiparkinsonian drug use. Participants who experienced fractures (n=124,655) were matched for age and sex with three randomly assigned controls (n=373,962). The authors found a dose-dependent increase in the risk of fracture related to levodopa use, either alone or combined with carbidopa and/or a COMT inhibitor.

### Strengths and limitations

This study has some strengths and limitations. As strength, we highlighted the detailed information collected on the drugs prescribed, showing prescription practices in routine consultations. As a limitation, we did not use a specific instrument to assess dyskinesia and its severity. It is critical to enhance dyskinesia diagnosis accuracy by educating patients and their families and utilize diaries or video recordings to track their frequency and severity. Also, we did not have data on locomotor function (balance and gait).

Individualized clinical assessment and review of drug prescribing in PD are practices that must be incorporated into neurological consultations. Health care providers play a relevant role in fall prevention in PD patients, particularly by identifying older adults experiencing dyskinesia and visual hallucinations. Also, patients taking amantadine and entacapone should be carefully assessed for fall risk. Prospective studies should investigate the possible role of these medications as risk factors for falls in PD patients.
